# 

**Published:** 1904-09

**Authors:** 


**This Journal and Atlanta Semi-weekly Journal one year for $1.25 cash in advance. No checks. Strictly to new subscribers.**

**This Journal and Atlanta Semiweekly Journal one year for $1.25 cash in advance. No checks. Strictly to new subscribers.**

the left hand, to affect in some degree the cortical center of the fifth nerve of the opposite side.

The method is so simple that it may be tried in a number of cases, and if without benefit it will be without harm.—*Med. Times.*

---

### ANNUAL SESSION MISSISSIPPI VALLEY MEDICAL ASSOCIATION.

The Thirtieth Annual Session of the Mississippi Valley Medical Association will be held at Cincinnati, Ohio, October 11, 12, 13, 1904, under the presidency of Dr. Hugh T. Patrick, of Chicago. The headquarters and meeting-places will be at the Grand Hotel.

The annual orations will be delivered by Dr. Wm. J. Mayo, of Rochester, Minn., in Surgery, and Dr. C. Travis Drenner, of Hot Springs, Ark., in Medicine.

Request for places upon the program, or information in regard to the meeting, can be had by addressing the secretary, Dr. Henry Enos Tuley, Louisville, Ky., or the assistant secretary, Dr. S. C. Stanton, Masonic Temple, Chicago, Ill.

The usual railroad rates will be in effect.

---

OF a list of fifty-six current deaths of physicians dying in the United States during the month of March, the average age was nearly sixty years—one reached ninety, seven were over eighty, and nineteen were more than three score and ten. This adds to the statistics we published a year or two ago to show that the mortality table so long in vogue, which class the doctors among the shortest lived of all the professional men, are certainly losing accuracy as applied to this country and this century. In the list above noted, four died under thirty-five of either tuberculosis or accidents, which could not be attributed to their occupation. These few materially lowered the average.—*Ex.*

---

***NOTICE.—This JOURNAL and THE INTERNATIONAL JOURNAL OF SURGERY one year for $1.00 cash, to new subscribers. Out-of-town checks not accepted unless 10c. is added for cost of collection. Strictly to new subscribers.***

**NOTICE.—This JOURNAL and THE INTERNATIONAL JOURNAL OF SURGERY one year for $1.00 cash, to new subscribers. No out-of-town Checks accepted unless 10c. is added for cost of cashing.**

**This Journal and Atlanta Semi-weekly Journal one year for $1.25, cash in advance. No checks. Strictly to new subscribers.**

